# Evaluation of the Differential Postbiotic Potential of *Shewanella putrefaciens* Pdp11 Cultured in Several Growing Conditions

**DOI:** 10.1007/s10126-023-10271-y

**Published:** 2023-12-28

**Authors:** Marta Domínguez-Maqueda, Jorge García-Márquez, Silvana T. Tapia-Paniagua, Carmen González-Fernández, Alberto Cuesta, Cristóbal Espinosa-Ruíz, María Ángeles Esteban, Francisco Javier Alarcón, María Carmen Balebona, Miguel Ángel Moriñigo

**Affiliations:** 1 Departamento de Microbiología, Facultad de Ciencias, Instituto Andaluz de Biotecnología y Desarrollo Azul (IBYDA), Universidad de Málaga, Ceimar-Universidad de Málaga, Málaga, Spain; 2 Departamento de Biología Celular e Histología, Facultad de Ciencias, Universidad de Murcia, Murcia, Spain; 3 Departamento de Biología y Geología, Universidad de Almería, Ceimar-Universidad de Almería, Almería, Spain

**Keywords:** *Shewanella putrefaciens* Pdp11, Postbiotic, Extracellular products, Growing conditions

## Abstract

The increased knowledge of functional foods has led to the development of a new generation of health products, including those containing probiotics and products derived from them. *Shewanella putrefaciens* Pdp11 (SpPdp11) is a strain described as a probiotic that exerts important beneficial effects on several farmed fish. However, the use of live probiotic cells in aquaculture has limitations such as uncertain survival and shelf life, which can limit their efficacy. In addition, its efficacy can vary across species and hosts. When probiotics are administered orally, their activity can be affected by the environment present in the host and by interactions with the intestinal microbiota. Furthermore, live cells can also produce undesired substances that may negatively impact the host as well as the risk of potential virulence reversion acquired such as antibiotic resistance. Therefore, new alternatives emerged such as postbiotics. Currently, there is no knowledge about the postbiotic potential of SpPdp11 in the aquaculture industry. Postbiotic refers to the use of bacterial metabolites, including extracellular products (ECPs), to improve host physiology. However, the production of postbiotic metabolites can be affected by various factors such as cultivation conditions, which can affect bacterial metabolism. Thus, the objective of this study was to evaluate the postbiotic potential of ECPs from SpPdp11 under different cultivation conditions, including culture media, temperature, growth phase, and salinity. We analyzed their hydrolytic, antibacterial, antiviral, and cytotoxic capacity on several fish cell lines. The results obtained have demonstrated how each ECP condition can exert a different hydrolytic profile, reduce the biofilm formation by bacterial pathogens relevant to fish, lower the titer of nervous necrosis virus (NNV), and exert a cytotoxic effect on different fish cell lines. In conclusion, the ECPs obtained from SpPdp11 have different capacities depending on the cultivation conditions used. These conditions must be considered in order to recover the maximum number of beneficial capacities or to choose the appropriate conditions for specific activities.

**Supplementary Information:**

The online version contains supplementary material available at 10.1007/s10126-023-10271-y.

## Introduction

The increased knowledge of functional foods has led to the development of a new generation of health products, including those containing probiotics (Palanivelu et al. 2022). Probiotics have been extensively researched for their beneficial effects on both marine and freshwater-farmed organisms, as summarized in numerous research studies (Chauhan and Singh 2019; El-Saadony et al. 2022; Austin and Sharifuzzaman 2022). However, despite their proven health benefits, the regulation and policy statements on the use of microorganisms make it difficult for non-lactic acid bacterial species to be generally recognized as safe (GRAS) (Sabahi et al. 2022).

Recent evidence suggests that bacterial viability is not necessary to achieve beneficial promoter effects. In this way, several studies have shown that probiotics dead cells or their derivatives metabolites can also have beneficial effects. These effects include the potential to inhibit pathogens (Danial et al. 2021), stimulate the immune system (Yeşilyurt et al. 2021), improve weight gain and specific growth rate, and enhance resistance against stress (Choudhury and Kamilya 2019), among others. In addition, our research group has observed some of those effects with the use of dead Pdp11 cells, which led to an increase in serum peroxidase content, natural hemolytic complement activity, phagocytic ability, and cytotoxic activity, as described by Díaz-Rosales et al. (2006). Recent studies by Domínguez-Maqueda et al. (2021) have shown no significant differences in growth, immunomodulation, or microbiota composition between live cells and heat-inactivated cell SpPdp11. In this way, widely attractive studies about postbiotics have emerged providing a potential opportunity in the field of functional foods (Moradi et al. 2021). In 2019, the International Scientific Association for Probiotics and Prebiotics (ISAPP) convened a panel that defined postbiotics as a “preparation of inanimate microorganisms and/or their components that confers a health benefit to the host.” Their application has been widely studied in human food, animal feed, and pharmaceuticals and has recently been investigated in the aquaculture industry (Cuevas-González et al. 2020).

Postbiotics have shown interesting properties associated with hydrolytic and antagonistic capabilities, inducing biological responses on host health and preventing intestinal diseases and microbial illness in farmed fish (Sudhakaran et al. 2022; Rad et al. 2022). Postbiotics have also demonstrated that they can improve the growth performance, composition, and function of gut microbiota, being a promising therapeutic agent to mitigate dysbiosis in aquaculture (Wu et al. 2020; Vargas-Albores et al. 2021). However, the production of postbiotics remains a challenge due to the limited knowledge on their methods of preparation and analysis, as well as the factors influencing their production (Garnier et al. 2019). Several factors affect the quantity and type of postbiotics products, including culture medium, bacterial treatment (heat inactivation, sonication, irradiation methods…), and growth phase (Moradi et al. 2021).

In this way, the production of organic acids (lactic acid, acetic acid, succinic acid, and formic acid) by different lactic acid bacteria (LAB) strains in anchovy infusion broth is significantly higher than in MRS broth (Ozcelik et al. 2016). In addition, modifying the composition of the culture medium can enhance the bacteriocin-inhibitory activity of postbiotics, as seen with the addition of extra glucose and yeast extract to a modified MRS medium used for *Lactobacillus plantarum* I-UL4 (Ooi et al. 2015). Dairy-derived ingredients, such as low-heat milk and milk permeate, have also been optimized, depending on incubation time and temperature, as fermentation media for *Lactobacillus* spp. to prepare postbiotic antifungal solutions (Garnier et al. 2019). In this way, aquafeed media could be an interesting cultivation condition that is as close to reality as possible. Therefore, optimizing the production of postbiotics could provide an opportunity for their use in different biotechnological applications, including the aquaculture/aquafeed industry.

*Shewanella putrefaciens* Pdp11 (SpPdp11) is a probiotic bacterium that was isolated from the skin of healthy gilthead seabream (*Sparus aurata*) (Chabrillón et al. 2005a) and has demonstrated numerous beneficial effects on farmed gilthead seabream and Senegalese sole (*Solea senegalensis*) over the years. These benefits include increased growth performance and body composition (García de la Banda et al. 2010; Tapia-Paniagua et al. 2014a), reduced mortality following pathogenic challenges (Tapia-Paniagua et al. 2015; Medina López 2018), stimulation of immunomodulatory capacity (Tapia-Paniagua et al. 2015; Domínguez-Maqueda et al. 2021), modulation of intestinal microbiota (Tapia-Paniagua et al. 2010, 2014b, 2015; Domínguez-Maqueda et al. 2021), and protective effects against oxidative stress (Vidal et al. 2016), among others. Despite these promising results, the postbiotic capabilities of SpPdp11 have not been analyzed. The in vitro study of metabolite activities provides valuable insights into the use of bacteria, while controlling culture conditions can open up new possibilities for optimizing their performance and biotechnological application.

The objective of the present study is to investigate how different cultivation conditions, such as temperature, incubation time, salinity, and media composition, affect the extracellular products (ECPs) secreted by SpPdp11. The ECPs will be evaluated for their hydrolytic, antibacterial, and antiviral activities. Furthermore, we have analyzed the impact of ECPs on the cytotoxicity of various fish cells. These findings will contribute to identifying the optimal conditions for ECP production, thereby enhancing their potential as postbiotics in the aquaculture industry.

## Material and Methods

### Bacterial Strain and Culture Conditions

*Shewanella putrefaciens* Pdp11 (SpPdp11) CECT 7627 was selected by its in vitro and in vivo ability to exert diverse beneficial effects as a probiotic on *S. aurata* and *S. senegalensis* specimens. SpPdp11 was grown as an axenic culture on tryptic soy agar supplemented with NaCl (1.5%) (TSAs) at 23 °C for 24 h. Then, one to two colonies were cultured on 50 mL of tryptic soy broth (Oxoid Ltd., Basingstoke, UK) supplemented with NaCl (1.5%) (TSBs) at 23 °C for 36 h (10^9^ UFC/mL, start of the stationary phase) on shaking at 80 rpm.

### Extracellular Product Extraction and Concentration

**Fig. 1:**
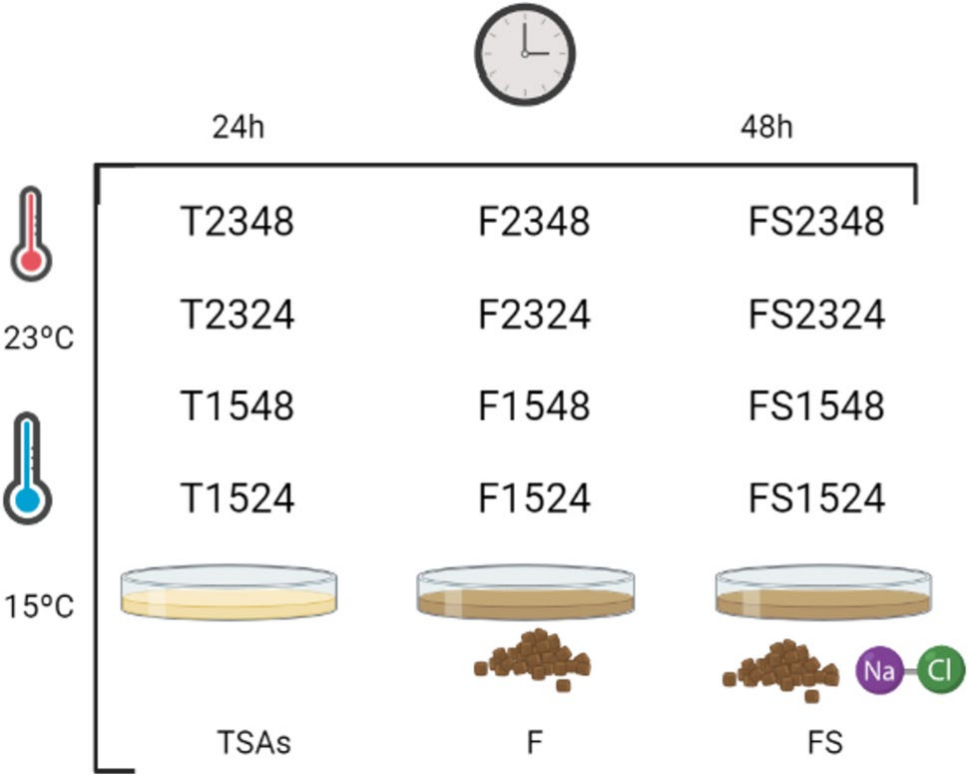
Different conditions for ECP extraction and nomenclature used in this experiment. Internal controls are not included but are named as their respective conditions but adding “control” at the end

Extracellular products (ECPs) from a solid medium were obtained by the collection through the cellophane plate technique described by Liu (1957). In brief, 1 mL of the SpPdp11 culture described above was spread over sterilized cellophane sheets placed on TSA plates (T media). Another 1 mL was spread over sterilized cellophane sheets placed on plates containing aquafeed (160 g/L) and agar (1.5%) supplemented with and without 1.5% NaCl (FS and F media, respectively). Aquafeed was kindly provided by Dr. Francisco Javier Alarcón (University of Almeria, Spain) (Table S1). Aquafeed media was included as a condition of culture because we aimed to study the ECPs secreted by the probiotic growing on the feed of farmed fish. The salinity of the aquafeed mostly from minerals is imperceptible, so the influence of salinity on the secretion of ECPs was also studied by supplementing the aquafeed media with 1.5% NaCl, because the probiotic SpPdp11 is a marine microorganism (Cuevas-González et al. 2020). The objective to evaluate the effect of temperature and the growth phase of SpPdp11 on the secretion of ECPs was approached by incubation of all inoculated plates at 15 °C and 23 °C, because they are temperatures applied for the farming of *S. aurata* (Georgakopoulou et al. 2010) and *S. senegalensis* (Campos et al. 2013), and for 24 h or 48 h (exponential or stationary stages, respectively). All different media, but without inoculation with SpPpd11, were incubated at the same conditions of temperature and time described previously and used as internal controls to check a possible background from the media. The different conditions assayed are summarized in Fig. 1.

Bacterial cells from different culture conditions and internal controls were harvested after 24 h and 48 h of incubation with 2 mL of sterile phosphate-buffered saline (PBS, pH 7.2), they were centrifuged (10,000 × *g*, 20 min, 4 °C), and the supernatants were filtered through 0.45- and 0.2-µm pore-size membrane filters (Merck Millipore, USA) to obtain only the ECPs. Controls were harvested in the same way. ECPs were also concentrated by Amicon Ultra centrifugal filters (10 K) (Merck Millipore, USA). Protein concentration was determined by Qubit Protein assay kits and the Qubit 2.0 (Thermo Fisher Scientific, USA). To ensure the absence of SpPdp11 growth and the presence of bacterial contamination, aliquots of ECP samples were cultured on TSAs and incubated for 24–48 h at 23 °C. They were kept at −80 °C until use.

### Characterization of Extracellular Product Activities

#### Hydrolytic Enzyme Production

A total of 19 enzymatic activities of the ECPs were evaluated with the API ZYM system (Ref #25 200, bioMerieux, Lyon, France) following the methodology indicated by the manufacturer. Sixty-five microliters of the concentrated ECP samples was added to each microcupule. The reaction was incubated for 4 h 30 min at 37 °C. Then, a drop of Zym A and Zym B reagents (bioMerieux) was added to each microcupule to develop chromogenic substrates. The results were interpreted following the commercial instructions by three independent observers.

In addition, extra activities such as phytase, tannase, and cellulase activities were assayed according to Kumar et al. (2010) on agar plates (1.5% agar) containing 1% w/v of Na-phytate (P-8810, Sigma), 2% w/v of tannic acid (P-403040, Sigma), and 1% w/v of carboxymethyl cellulose (CMC) (C-5678, Sigma), respectively. Those are known for their anti-nutritional properties, mostly associated with vegetal compounds. Furthermore, protease, gelatinase, lipase, and amylase activities were assayed according to Chabrillón et al. (2005b) on agar plates containing 2% w/v of skim milk (Pirinea, Spain), 1% w/v of gelatine (Oxoid, UK), 1% w/v of Tween 80 (Panreac, EEUU), and 4% w/v of starch (Labkem, USA), respectively. In all cases, 50 µL ECP samples (0.5 µg protein/µL) and internal controls were inoculated into 6-mm-diameter wells made in the plates and incubated at 23 °C for 24–48 h. The plates were observed for the presence of a clear zone around the wells. Hydrolytic activities against starch and carboxymethyl cellulose (CMC) were indicated by a clear zone around the colonies after flooding the plates with Lugol and Congo red solution 0.1% w/v, respectively. Fifty microliters of PBS was used as a negative control and 50 µL *Vibrio proteolyticus* cells (10^8^ ufc/mL) as a positive control. The absence of a clear zone was interpreted as the absence of activity. The lowest ECP concentration with a clear zone around the well was designated as the minimum concentration of each activity. Each ECP condition was tested in triplicate, and each experiment was repeated twice.

### Antagonistic Activity Against Pathogens

#### Antibacterial Activity

Antibacterial activity was performed using the agar well diffusion assay as described by García-Márquez et al. (2021). Some strains were obtained from the Spanish Collection of Type Cultures (CECT). Fish pathogenic bacterial strains *Aeromonas hydrophila* (Arijo et al. 2005), *Vibrio harveyi* 16/00 (Arijo et al. 2005), *Vibrio anguillarum* (CECT 522), *Photobacterium damselae* subsp. *damselae* (CECT 626), and *P. damselae* subsp. *piscicida* (Díaz-Rosales et al. 2003; Arijo et al. 2005) were cultured on TSA plates at 23 °C for 24 h. In addition, *Tenacibaculum maritimum* (CECT 4296), *T. soleae* (CECT 7292), and *T. gallaicum* (CECT 7122) were cultured on *Flexibacter maritimus* medium (FMM) (Pazos et al. 1996) plates supplemented with agar (1.5%) at 28 °C for 48 h. Standardized cultures adjusted to OD_600nm_ ~0.1 were evenly spread onto the surface of the TSAs or FMM plates using sterile swab sticks. Six wells (6 mm diameter) were made on each plate with the wide end of a sterile glass Pasteur pipette. Fifty microliters of adjusted ECP samples (0.5 µg protein/µL) to a final concentration of 25 µg protein and internal controls was added to each well. Fifty microliters of TSBs or FMM and PBS was used as a negative control and 50 µL *Vibrio proteolyticus* cells (10^8^ ufc/mL) as a positive control. Plates were incubated for 24–48 h at 23 °C or 28 °C depending on the optimal time of incubation and temperature for each pathogen.

The plates were observed for the presence of inhibition of bacterial growth, which was indicated by a clear zone around the wells. The size of the zones of inhibition was measured, and the antibacterial activity was expressed in terms of the average diameter of the zone inhibition in centimeters. The absence of a zone inhibition was interpreted as the absence of bacterial growth or antibacterial activity. Each ECP condition was tested in triplicate, and each experiment was repeated twice.

#### Biofilm Inhibition Assay

Biofilm formation assay was performed by crystal violet (CV) staining, as described by Vivas et al. (2008), with modifications (Acosta et al. 2021). *T. maritimum*, *T. soleae*, and *T. gallaicum* were cultured in FMM plates supplemented with agar (1.5%) at 28 °C for 48 h and *V. anguillarum* and *A. hydrophila* in TSA plates at 23 °C for 24 h. One colony of each strain was placed in their respective liquid media (FMM for *T. maritimum*, *T. soleae*, and *T. gallaicum* and TSBs for *V. anguillarum* and *A. hydrophila*) and adjusted to OD_595nm_ ~0.1. Then, 20 µL of the bacterial suspensions was pipetted into flat-bottom polystyrene 96-well plates (#D51588, Sarstedt, Nümbrecht, Germany) and filled up to 200 µL of FMM or TSBs. To determine the ability to form a biofilm, microplates were incubated under static conditions at 28 °C for 48 h for *T. maritimum*, *T. soleae*, and *T. gallaicum* and 23 °C for 24 h for *V. anguillarum* and *A. hydrophila*, allowing cells to attach to the polystyrene. Simultaneously, to compare and determine the inhibition of biofilm formation, 20 µL of pathogenic bacterial suspensions was pipetted again, and microplates wells were filled up to a final volume of 200 µL by adding 90 µL of TSBs or FMM double concentrated and 90 µL of quantities known (~50 µg) of ECP treatments. ECPs were added at the beginning (0 h) of incubation, and subsequent quantitative biofilm formation was measured after incubation of 24 h for *V. anguillarum* and *A. hydrophila* and 48 h for *T. maritimum*, *T. soleae*, and *T. gallaicum*. After incubation, the liquid medium above the biofilm layers was removed by inversion, and the wells were washed three times with PBS. The resulting biofilm layers were fixed for 20 min with 200 μL of 99% methanol per well. Then, microplates were air-dried and stained for 15 min with 200 μL of CV solution (0.1% w/v solution) per well. Excess staining was removed by washing three times with distilled water. The plates were air-dried, and the CV was removed with 200 μL of 33% acetic acid. The plates were left for 3 min at room temperature in an orbital plate shaker at 200 rpm to facilitate the removal of colorant. Next, 150 μL from each well was transferred to a flat-bottom 96-microplate, and the amount of dye, proportional to the number of bacteria adhered, was quantified at OD_595nm_ in a plate reader (Multiskan FC, Thermo Fisher). Growth performance was assayed at the same time. Each value was subtracted from the control cell values, which only contained the culture medium. These experiments were carried out in triplicates, with five wells (*n* = 5) per strain in each assay.

### Antiviral Activity

The antiviral activity was tested against nervous necrosis virus (NNV, RGNNV genotype, strain It/411/96), produced and titrated in the E−11 cell line, a clone of the striped snakehead fish (*Channa striata*) cell line, at 25 °C. E−11 cells were grown at 25 °C in Leibovitz’s L-15 medium (Gibco) supplemented with 10% fetal bovine serum (FBS) (Gibco), 2 mM L-glutamine (Gibco), 100 i.u./mL penicillin (Gibco), 100 μg/mL streptomycin (Gibco), and 0.25 μg/mL amphotericin B (Gibco) using Falcon Primaria cell culture flasks (Becton Dickinson). Cells were subcultured by trypsin routine methods. Viral stocks were generated until the cytopathic effect (CPE) was extensive when supernatants were harvested and centrifuged to eliminate cell debris. Virus stock was titrated using their respective cell hosts and temperatures in 96-well plates and expressed as the viral dilution infecting 50% of the cell cultures (TCID50), before being used in the in vitro experiments.

To evaluate the antiviral activity, 1 or 15 µL of culture medium (controls) or ECP samples from each condition studied were incubated with NNV aliquots (1:20 or 15:20 of ECPs-to-final volume) for 24 h at 25 °C (León et al. 2020). After this, virus solutions were used for titration in triplicate. For this, E−11 cells were seeded in 96-well plates reaching the 60–80% confluence the next day. Then, the medium was removed, and cells were incubated with tenfold dilutions of the samples in a temperate medium without FBS for 2 h. Afterward, the supernatant was discarded, and cells were incubated with 100 μL of medium with 2% FBS. Cultures were daily observed under a phase microscope, and the cytopathic effect was monitored for 7–10 days. Finally, the TCID50/mL was calculated for each sample and represented. Control samples (medium+NNV) represent 100% viral activity or 0% antiviral activity. The results are expressed as % viral titer with respect to the untreated virus or controls. To discard that E–11 cells dye by the ECPs, and not by NNV, cells were also incubated with ECPs alone. Data are representative of two independent experiments.

### Cytotoxic Effect

#### Cell Culture

The established SAF-1 (ECACC n°00122301) cell line obtained from fibroblast cells of seabream was seeded in 25 cm^2^ plastic tissue culture flasks (Nunc, Germany) cultured in L-15 Leibowitz medium, supplemented with 10% FBS, 2 mM/L L-glutamine, 100 i.u./mL penicillin, and 100 μg/L streptomycin. Cells were grown at 25 °C under a humidified atmosphere (85% humidity). The DLB-1 (CVCL_HG31) cell line obtained from the European sea bass brain was used (Morcillo et al. 2017). Cell monolayers were grown at 25 °C in L-15 Leibovitz medium containing 0.16% NaCl, 15% FBS, 20 mM HEPES (Thermo Fisher Scientific), 2 mM/L glutamine, 100 i.u./mL penicillin, and 100 μg/mL streptomycin. The cells were cultured at 25 °C in an incubator with an atmosphere with 85% relative humidity. The FuB-1 (CVCL_YJ47) cell line obtained from *Fundulus heteroclitus* brain was also grown at 25 °C in a humidified atmosphere (85% humidity) in L-15 Leibowitz medium supplemented with 15% FBS, 2 mM/L glutamine, 100 μg/mL streptomycin, 100 i.u./mL penicillin, and 10 mM HEPES (Ruiz-Palacios et al. 2020). Finally, the established cell line PLHC-1 (ATCC^®^ CRL2406^™^) derived from a hepatocellular carcinoma of *Poeciliopsis lucida* was seeded into 25 cm^2^ plastic tissue culture flasks in Eagle’s minimum essential medium (EMEM) (Sigma) with 2 mM/L L-glutamine and Eagle salts adjusted to contain 1.5 g/L sodium bicarbonate, 0.1 mM non-essential amino acids, 1.0 mM sodium pyruvate, 5% FBS, 100 i.u./mL penicillin, and 100 mg/mL streptomycin. The cells were grown at 30 °C in a humidified atmosphere (85% humidity) with 5% CO_2_.

#### MTT Assay

A cytotoxicity assay of each cell type was performed in five replicates with each concentration of each extract. When cell lines reached approximately 80% confluence, the cells were detached from the flasks with trypsin according to the standard trypsinization methods (0.25% trypsin for SAF-1, DLB-1, and FuB-1 cells, and 0.05% trypsin for PLHC-1 cells), and aliquots of 100 μL containing 50,000 cells/well were dispensed into 96-well tissue culture plates and incubated (24 h, at the optimal temperature for each cell line). This cell concentration was previously determined so that satisfactory absorbance values would be obtained in the cytotoxic assay and to avoid cell overgrowth. After that, the culture medium was replaced by 100 μL/well of the ECP extracts containing 0.75, 1, and 1.5 mg/mL of proteins. Control samples received the same volume of culture medium. Cells were incubated for 24 h, and then, viability was determined by the MTT assay (Stevens et al. 1991), which is based on the reduction of the yellow soluble tetrazolium salt (3-(4,5-dimethylthiazol-2-yl)-2,5- diphenyltetrazolium bromide) (MTT, Sigma-Aldrich) to a blue, insoluble formazan product by mitochondrial succinate dehydrogenase. Cells were washed with PBS, and 200 μL of MTT (1 mg/mL) was added per well. After 4 h of incubation, the cells were washed again, and the formazan crystals were solubilized with 100 μL/well of DMSO. The plates were shaken (5 min, 100 rpm) under dark conditions, and absorbance was determined at 570 nm and 690 nm in a microplate reader.

### Statistical Analyses

Statistical analyses were conducted using IBM SPSS Statistics 22.0. All data were analyzed in the same way. Firstly, normality and homogeneity of variance of the data were determined by using Shapiro-Wilk’s and Levene’s tests, respectively. Differences were statistically analyzed by one-way analysis of variance (ANOVA) with post hoc tests. Tukey test was performed when the data met normality and homoscedasticity while the Games-Howell test was performed when data met normality but no homoscedasticity. Non-normally distributed data, which did not meet normality nor homoscedasticity, were analyzed by the non-parametric Kruskal-Wallis with the Bonferroni correction test, followed by a multiple comparison test. Statistical significance was set for *p* ≤ 0.05.

## Results

### Hydrolytic Activities of ECPs

The results of enzymatic activities evaluated using the API ZYM are summarized in Table 1. ECPs obtained from TSAs showed all 19 enzymatic activities regardless of the temperature and time of incubation. On the other hand, ECPs recovered from the aquafeed medium (F) were dependent on the time of incubation since 19 and 18 activities were detected when ECPs were recovered at 15 °C and 23 °C for 24 h, respectively, and 11 and 8 activities at 15 °C and 23 °C for 48 h, respectively. In addition, NaCl supplementation of the aquafeed medium (FS) reduced the number of enzymatic activities detected in comparison to those not supplemented, especially at 15 °C. Generally, the activities decrease at 48 h in comparison with 24 h, being the activities less detected lipase (C14), arylamidases, trypsin, α-chymotrypsin, and enzymes involved in the hydrolysis of carbohydrates such as α- and β-galactosidase, β-glucuronidase, α-glucosidase, and β-fucosidase. On the other hand, the aquafeed media improve the alkaline phosphatase activity regardless of the temperature or time of incubation, while ECPs from aquafeed at 23 °C decrease the esterase and ester lipase activity. In addition, the N-acetyl-β-glucosaminidase activity was highly stimulated by FS2324 and T2324 treatments. Internal controls did not show an enzymatic reaction in any case.

**Table 1:**
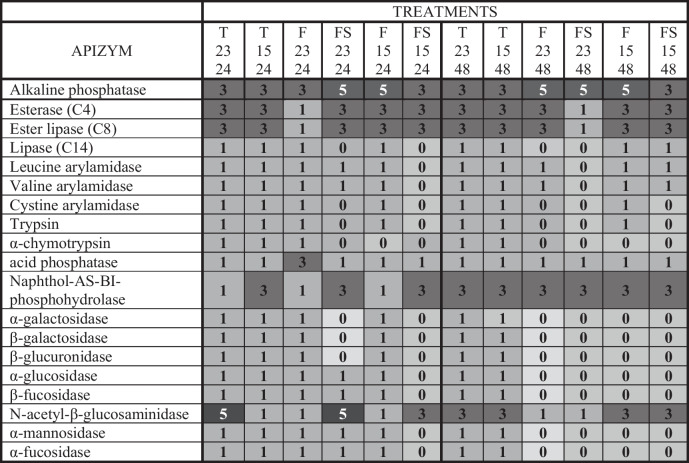
Hydrolytic activities produced by ECP samples (µg protein), extracted from different cultivation conditions, of the *S. putrefaciens* Pdp11 strain

Values are averages of three API ZYM readings expressed as color intensity of enzymatic reactions (0, no intensity; 1, low intensity; 3, moderate intensity; 5, high intensity)

On the other hand, Table 2 represents the minimum concentration of ECPs that exerts hydrolytic activities of nutritional and anti-nutritional compounds. All the conditions studied, except T1524, were able to hydrolyze gelatine, casein, and Tween 80 at 24 and 48 h. Furthermore, only FS2324 and FS2348 were positive for starch hydrolysis. As above, in general, ECPs recovered at 24 h of incubation showed higher activities than those at 48 h, regardless of temperature and substrate, except for gelatin (T2324, T1548) and lipase (T2348) activities. In any case, there was hydrolysis of cellulose, tannins, and phytate. Internal controls did not show hydrolytic activities.

**Table 2:** Hydrolytic activities produced by ECP samples (µg protein), extracted from different cultivation conditions, of the *S. putrefaciens* Pdp11 strain

TREATMENTS												
	T	T	F	FS	F	FS	T	T	F	FS	F	FS
	23	15	23	23	15	15	23	15	23	23	15	15
	24	24	24	24	24	24	48	48	48	48	48	48
Amylase	ND	ND	ND	22.50	ND	ND	ND	ND	ND	22.50	ND	ND
Cellulase	ND	ND	ND	ND	ND	ND	ND	ND	ND	ND	ND	ND
Gelatinase	10.67	2.67	14.00	1.83	6.58	0.93	5.17	0.96	20.73	1.83	2.17	2.17
Caseinase	2.53	ND	24.35	1.83	1.70	3.23	20.73	5.17	14.00	1.83	2.17	2.17
Lipase	1.85	ND	7.76	6.58	1.67	3.28	2.70	0.48	3.28	6.58	2.50	1.07
Phytate	ND	ND	ND	ND	ND	ND	ND	ND	ND	ND	ND	ND
Tannins	ND	ND	ND	ND	ND	ND	ND	ND	ND	ND	ND	ND

Minimum protein concentration (µg protein)

*ND* not detected

### Antimicrobial Activity of ECPs

#### Antibacterial Activity

Regardless of the condition, the different ECPs and their internal controls did not exhibit any antibacterial activity (data not shown).

#### Effect on Biofilm Formation

**Fig. 2:**
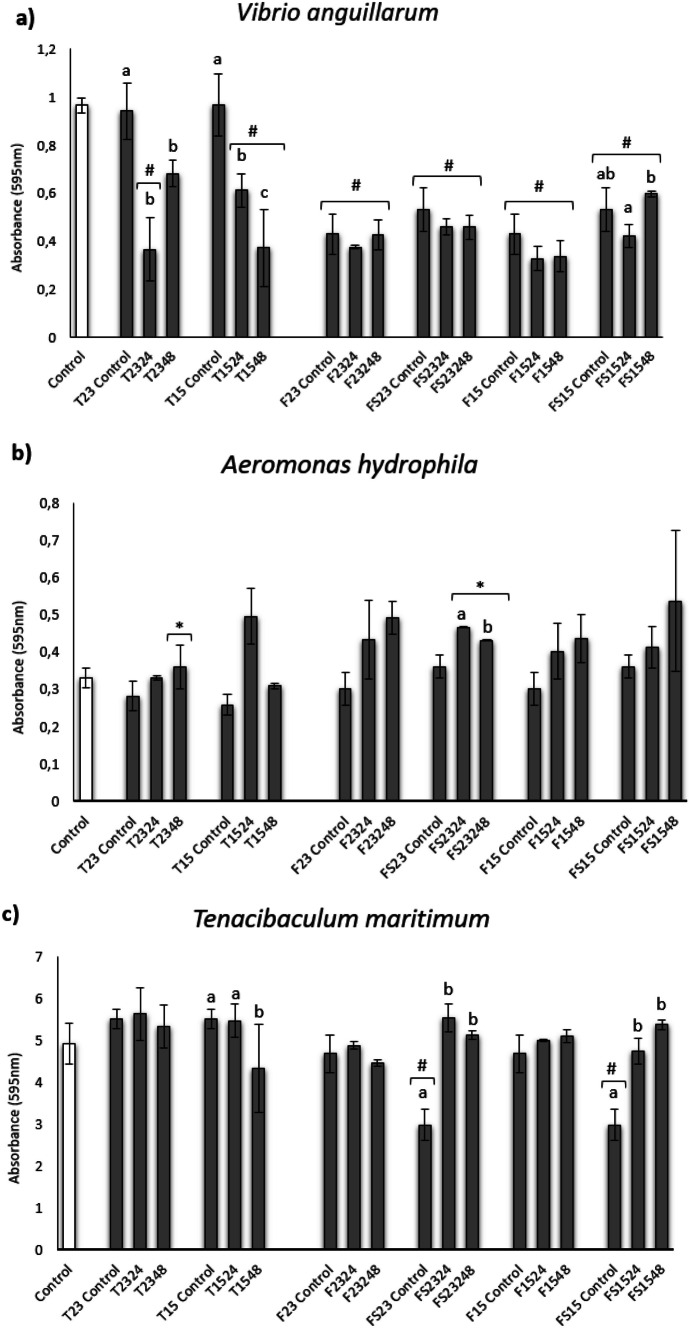

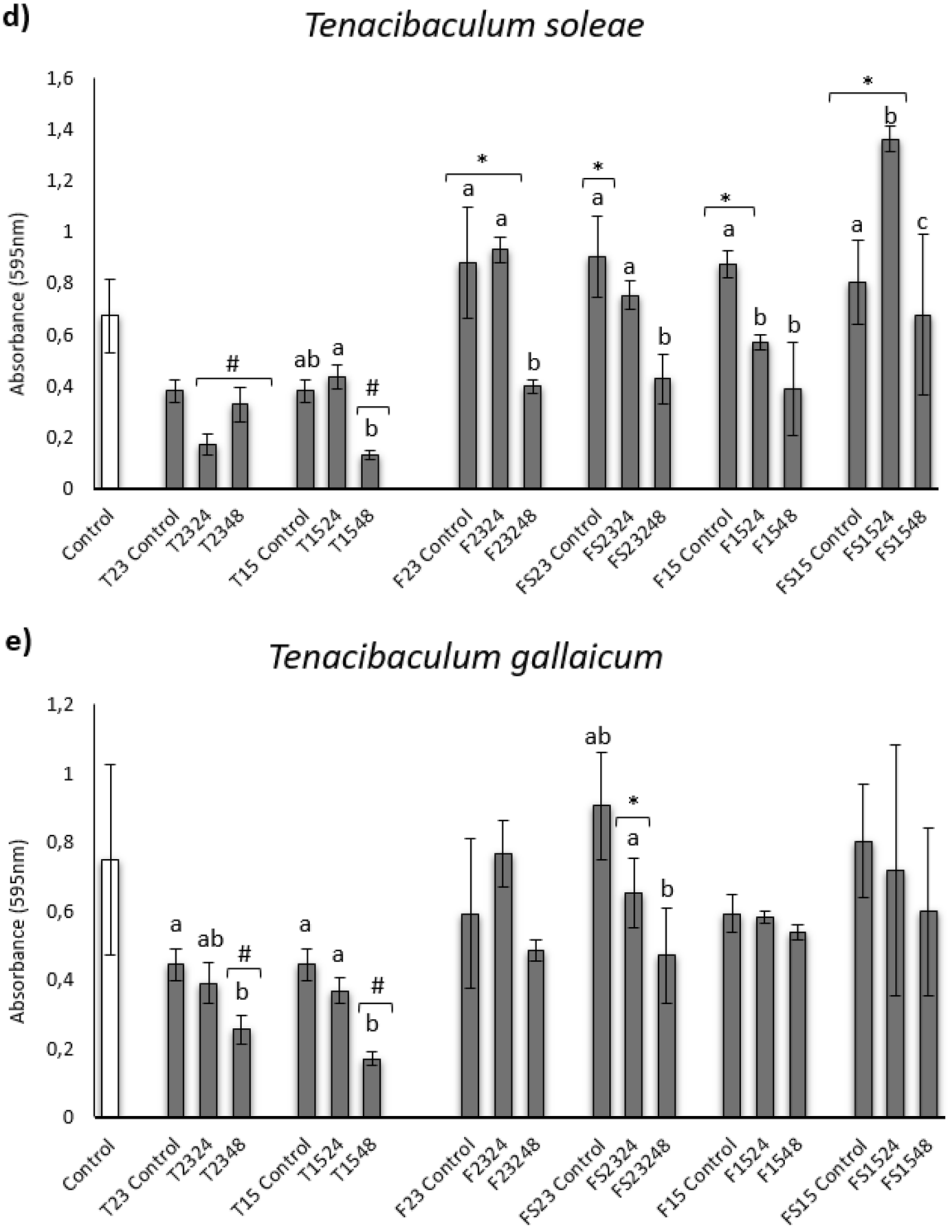
Biofilm formation inhibition of *Vibrio anguillarum* (**a**), *Aeromonas hydrophila* (**b**), *Tenacibaculum maritimum* (**c**), *Tenacibaculum soleae* (**d**), and *Tenacibaculum gallaicum* (**e**) after 24 h (**a**, **b**) and 48 h (**c**–**e**) of incubation of the different bacteria with ECP samples (µg protein/µL), extracted from different growing conditions of the *S. putrefaciens* Pdp11 strain. White bars represent the biofilm formation of bacteria (control group). The results are representative of three independent experiments and are expressed as mean ± SD (*n* = 5). Square brackets with # or * indicate reduction and proliferation, respectively, of biofilm formation between treatments and bacterial control (*p* < 0.05). Different letters indicate significant differences between treatments and internal controls (*p* < 0.05)

The effect of different ECPs obtained and the internal controls on the biofilm formation of several fish pathogens are shown in Fig. 2. All ECPs obtained from the TSA medium showed a significant reduction of the biofilm, showing the treatments T2324 and T1548 the highest reduction (Fig. 2a). The results showed a reduction on the biofilm formation of *V. anguillarum* by the internal control corresponding to F media, with the ECPs collected from FS1524 being the only ones with the ability to reduce the formation of biofilm (Fig. 2a). Anyway, all these conditions similarly affect the biofilm formation regardless of the temperature, time of incubation, and salinity. On the other hand, none of the ECPs were capable of reducing the biofilm formation of *A. hydrophila*. On the contrary, there is a significant increase in the case of T2348, FS2324, and FS2348 treatments (Fig. 2b). Regarding the different strains of *Tenacibaculum* assayed, *T. maritimum* biofilm formation was not significantly reduced by any ECPs (Fig. 2c). Biofilm formation by *T. soleae* and *T. gallaicum* was significantly reduced by ECPs obtained from TSAs at 48 h regardless of incubation temperature and from T2324 in the case of *T. soleae* (Fig. 2d, e). In contrast, all internal controls and ECPs obtained from FS1524 and F2324 showed a significant increase in *T. soleae* biofilm development (Fig. 2d), while ECPs from F2324 showed a statistical increase in *T. gallaicum* biofilm formation (Fig. 2e).

### Antiviral Activity

**Fig. 3:**
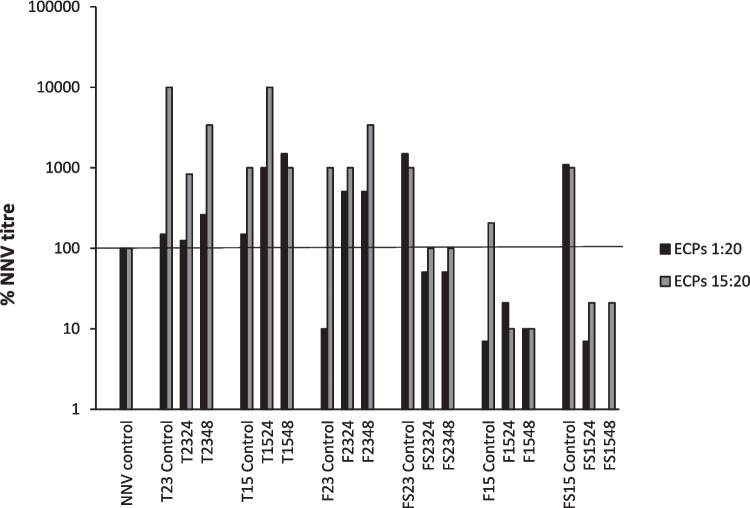
Antiviral activity of the ECPs. ECPs were incubated for 24 h with NNV at a final concentration of 1:20 or 15:20. Afterwards, NNV was titrated in the E−11 cell line. Control samples consisted of virus incubated with the same amount of medium. Viral titer respect to the control is represented. Data are representative of two independent assays

Figure 3 shows that all ECPs and internal controls from the TSA medium increased the viral titer, some of them by more than 1 log regardless of the temperature and time of incubation. On the contrary, ECPs extracted from F and FS media showed more variability. Internal controls from F/FS media tend to increase the NNV titer except for F23 and F15 control. A clear effect of incubation temperature was observed regarding the antiviral activity of ECPs obtained from F and FS media, because while ECPs obtained at 23 °C increased or maintained the NNV titer at 15 °C which was observed to be an important antiviral activity regardless of the media or time of incubation, especially in the case of the highest dose of ECPs.

### Cytotoxicity of ECPs

**Fig. 4:**
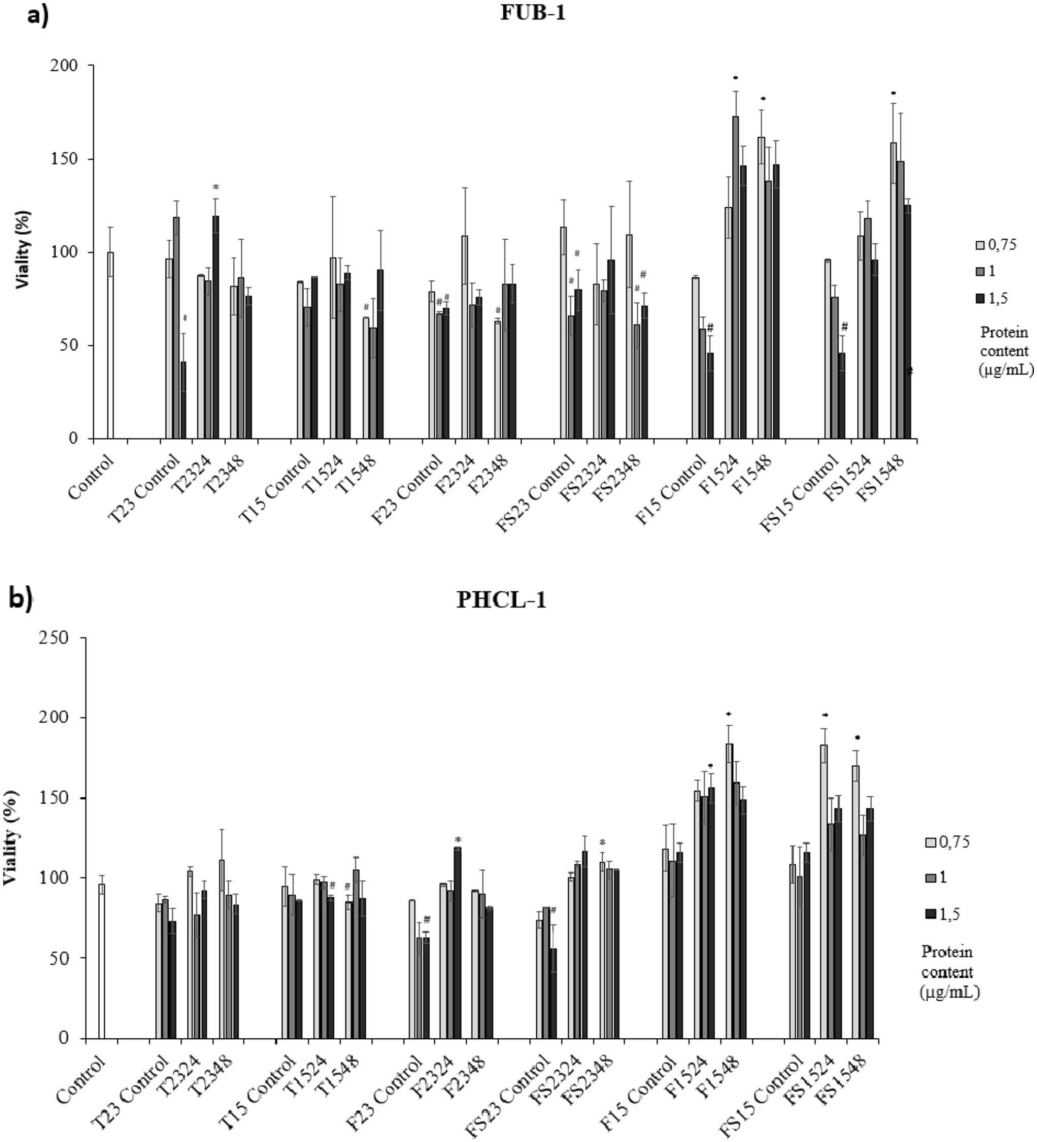

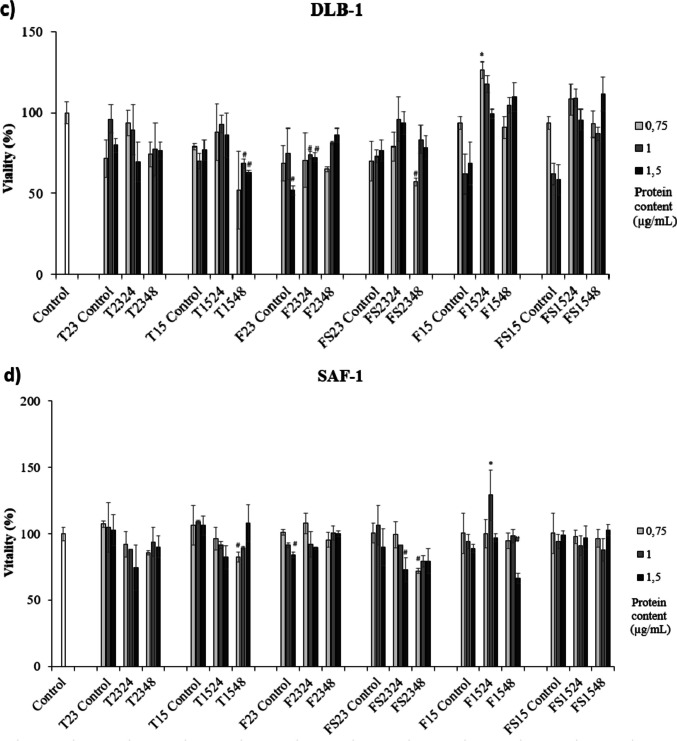
Cytotoxic effect produced by ECPs of *S. putrefaciens* Pdp11 on various cell lines. **a** Brain cell line (FuB-1) from mummichog (*Fundulus heteroclitus*), **b** fish hepatoma cell line (PLHC-1) (*Poeciliopsis lucida*), **c** brain cell line (DLB-1) from European sea bass (*Dicentrarchus labrax*), and **d** fibroblast cell line (SAF-1) (*Sparus aurata*). The increase in viability was detected after 24 h of incubation. The concentrations of ECPs tested on all cells were 0.75, 1, and 1.5 µg/mL. The values reported are the means of three replicates. Prime symbol (#) or asterisks (*) indicate reduction and proliferation, respectively, of cell viability (*p* < 0.05)

Most of the ECPs were not significantly cytotoxic in any of the cell lines tested (Fig. 4). However, there were some conditions, such as T1548 and F23 control, which showed a significant cytotoxic effect on most cell lines (Fig. 4). On the other hand, the ECPs obtained from F and FS media were lower cytotoxic at higher doses than those recovered from the TSA medium. Moreover, ECPs obtained from F and FS media at 15 °C were lower cytotoxic than those at 23 °C. In this way, ECPs from F1524 were the best and non-cytotoxic condition in all cell lines assayed (Fig. 4). In the case of FUB-1 cells, cytotoxicity was significantly increased compared to the rest of the cell lines, independently of the condition except for those ECPs obtained from F and FS media at 15 °C, avoiding internal controls (Fig. 4a). Most of the ECPs obtained from F and FS media were non-cytotoxic of the PLHC-1 cell line, especially at 15 °C (Fig. 4b). Cytotoxicity on the DLB-1 cell line was not very pronounced except for ECP conditions T1548 and F2324, both at 1 and 1.5 µg/mL, F23 control at 1.5 µg/mL, and FS2348 at 0.75 µg/mL (Fig. 4c). Specifically, the viability of SAF-1 cells was the least affected (Fig. 4d).

## Discussion

Probiotics are considered a promising and useful alternative for disease control in aquaculture, and their study has been constant. In this sense, numerous beneficial characteristics have been described for SpPdp11 as viable and heat-inactivated cells in both in vivo and in vitro studies (Cámara-Ruiz et al. 2020). However, the potential capabilities that could be present in their ECPs and, consequently, a potential postbiotic application of the probiotic have not been evaluated until now.

### Hydrolytic Activities of ECPs

Probiotic derivatives are promising sources of enzymes with potential biotechnological applications (Yadav et al. 2020). In the farmed fish industry, postbiotics can be used as food supplements to improve the transformation and/or utilization of different energy sources (e.g., carbon, lipids, proteins) in animals (Vera-Gargallo and Ventosa 2018). If the necessary enzymes are not present in digestion, a loss of the consumed food energy of between 20 and 80% can occur (Debnath et al. 2007). Therefore, the addition of enzymes could be beneficial in optimizing nutrient utilization in farmed fish. In this study, we found that the enzymatic activity of the ECPs of SpPdp11 varied depending on the media in which their cells were cultured. Specifically, the ECPs recovered from T and F media, regardless of temperature and time of incubation, had the highest number of enzymatic activities at both 15 °C and 23 °C for 24 h. It is possible that after 48 h, bacterial cultures begin to enter the stationary phase, and the scarcity of nutrients limits enzyme production (Kim et al. 2020).

The presence of N-acetyl-beta-glucosaminidase activity in the majority of the ECPs obtained after 48 h is remarkable. This enzyme has an antibacterial capacity, and perhaps, its synthesis may be a desperate attempt by the cells to obtain nutrients when they are scarce in the medium. Moreover, this enzyme is involved in the degradation of chitin, a linear polysaccharide of 1, 4-β-linked N-Acetyl glucosamine (GlcNAc), which is the second most abundant renewable source in nature after cellulose (Loughran and Emerling 2022). This point is relevant because insects are a novel feed source for animal nutrition due to their high nutritional value with a reduced environmental impact, but they also contain chitin, a complex polysaccharide which is resistant to digestion in the gut of most animals (Gasco et al. 2021). Chitinases are the enzymes that can hydrolyze chitin, but sometimes, the gut of animals lacks the necessary activity to hydrolyze it, which can affect nutrient absorption, resulting in poor growth, lower feed efficiency, reduced performance in animals, increased susceptibility to pathogen infections, and alteration of gut microbiota (Sharma et al. 2023). Further research is needed to understand how fish respond to chitin and how the gut microbiota of fish can be manipulated to improve chitin digestion. In addition, alternative strategies for breaking down chitin, such as the use of exogenous chitinase enzymes, may also need to be explored. This fact could be an improvement in obtaining sources of alternative nutrients.

Other notable activities detected in ECPs recovered at 48 h are the naphthol-AS-BI-phosphohydrolase and the alkaline phosphatase. The first is considered an essential enzyme involved in the digestive process to release phosphorylated groups (Diez-Gutiérrez et al. 2022), while the second is usually produced by enterocytes to keep the gastrointestinal (GI) tract (and systemic) inflammation under control (Lallès 2020). Proteolytic enzymes are crucial in many physiological processes, including digestion, absorption, immunological defense, cell growth, cell death, and apoptosis (Kaur and Singh 2022), with their activity seeming to be affected by salinity. Higher salinity concentrations were found to decrease the activity of enzymes such as trypsin and chymotrypsin. These enzymes’ potential use could be explored in future aquaculture or farmed animal applications.

The uptake of dietary lipids mainly depends on carboxyl ester hydrolases and lipases, enzymes that were evaluated in the APIZYM profile. According to Diez-Gutiérrez et al. (2022), both lipase and esterase activity of probiotics participate beneficially in the GI tract by increasing the absorption of nutrients, improving metabolism, and maintaining GI structure. However, the lipase (C14, myristic acid, a saturated fatty acid frequently present in food (Takato et al. 2017)) was found to be poorly detected when the ECPs were recovered from the aquafeed medium. This may be attributed to the presence of fatty acids in the commercial feed that were already available to SpPdp11 during its growth. Previous studies have shown that the presence of dietary fat can increase the expression of genes involved in lipid metabolism and transport in the intestines of fish and other animals (Kemski et al. 2020; Araújo et al. 2022). This could result in an increase in the production of digestive enzymes, such as lipases, that are needed to break down the fatty acids in the diet. However, the efficacy of this activity depends on the specificity of the enzyme, which can vary based on the substrates.

Regarding specific enzymatic activities, lipase, caseinase, and gelatinase were the most active enzymes of the ECPs. The hydrolyzation of Tween 80, an unsaturated fatty acid used as a food additive (up to 1%) (Nielsen et al. 2016), was observed in ECPs except for T1524, due to the absence of lipase activity. This contrasts with the results obtained in API ZYM, which could be attributed to the supplementation of NaCl in the plates, a molecule known to intensify the lipolytic activity (Carrazco-Palafox et al. 2018). The potential to degrade proteins by ECPs is demonstrated by the hydrolysis of casein and gelatin by ECPs of SpPdp11. This hydrolysis of proteins could also promote positive effects on fish performance and protein digestibility of some carnivorous fish (Hamre et al. 2013). Finally, amylase activity was only detected in ECPs obtained from the FS medium at 23 °C (FS2324 and FS2348). Amylase activity allows the degradation of carbohydrates, improving the decomposition of sugars in the intestine (Nkhata et al. 2018). On the other hand, none of the ECPs tested showed the capability to degrade any of the anti-nutritional factors tested. These findings suggest that the observed abilities of the ECPs of this probiotic could be related to the beneficial effect exerted by SpPdp11 on the growth of *S. senegalensis* specimens, as reported in several previous studies (Sáenz de Rodrigáñez et al. 2009; García de la Banda et al. 2010).

The production and release of hydrolytic enzymes by SpPdp11 were found to be influenced by the culture conditions and particularly by the supplementation of NaCl and the capacity of SpPdp11 to produce activities useful as postbiotics. The concentration of salinity can affect microbial growth by affecting the osmotic pressure inside and outside cells, which can impact the activity of biological enzymes (Li et al. 2022). These results highlight the key role of culture conditions in determining the hydrolytic potential of ECPs of SpPdp11 and its optimization for the application of ECPs as postbiotics and to obtain the maximum activity in terms of nutrient assimilation.

### Antimicrobial Activity of ECPs

#### Antibacterial Effect

Another interesting application/feature of postbiotics is their antibacterial activity against pathogens (Rajoka et al. 2018; Abdalla et al. 2021). However, none of the ECPs tested in this study inhibited any of the pathogens assayed, in contrast to the results reported by Chabrillón et al. (2005a), who observed the ability of live cells of SpPdp11 to inhibit the growth of fish pathogens, such as *Photobacterium damselae* subsp. *piscicida*. In addition, this fact could also be contradicted due to the presence of N-acetyl-beta-glucosaminidase, gelatinase, and lipase activity that could affect bacterial surfaces and destroy them. These activities have been detected in probiotic strains of lactic acid bacteria (LAB) regarding the digestive process, as well as their antagonistic and lytic activities against other pathogen microorganisms (Hossain et al. 2020). However, it is also possible to think that inhibition can be a success that occurs between living cells and their interactions, and the role of ECPs is not enough due to the dose or types of enzymes for the activities assayed in this study. Therefore, more research on the mechanisms involved in bacterial inhibition is essential.

#### Effect on Biofilm Formation

The antibacterial capacity can also be related to the formation of pathogenic biofilm inhibition. Bacterial biofilms are clusters of bacteria that are attached to a surface and/or to each other and embedded in a self-produced matrix containing proteins, polysaccharides, and extracellular DNA (Vestby et al. 2020). Because of this, microorganisms can make themselves more resistant to physical, biological, and chemical factors such as antimicrobial therapy (Vestby et al. 2020). For example, exopolysaccharide production is essential for the pathogenicity of *Vibrio anguillarum* biofilm formation (Croxatto et al. 2007; Li et al. 2014), while biofilm formation in *Tenacibaculum* species is considered a mechanism for environmental persistence and bacterial transmission (Levipan et al. 2019a, b; Dong et al. 2022). Several studies have reported that the effect of proteases can reduce biofilm formation (Mitrofanova et al. 2017; Saggu et al. 2019). In this context, only ECPs produced by SpPdp11 in TSAs reduced the *V. anguillarum* biofilm formation, and they showed a high number of hydrolytic activities, which could be involved in the reduction of the *V. anguillarum* biofilm. These ECPs also showed higher activities related to proteases than those obtained from F and FS media, such as gelatinase and especially caseinase, both at 15 °C and 23 °C. However, F/FS media generally promotes the biofilm of the *Tenacibaculum* strains, mainly in *T. soleae*. This effect may also result from the interaction between bacterial molecules present in ECPs and their implications in quorum sensing (QS) and/or quorum quenching (QQ) mechanisms, which can promote (e.g., *T. soleae*) or inhibit (e.g., *V. anguillarum*) the biofilm formation, respectively (Khan et al. 2019; Paluch et al. 2020). These findings highlight the importance of selecting the appropriate culture medium for obtaining postbiotics. Moreover, the salinity would play an important role, as reported by John et al. (2019), in which an increase in NaCl concentration can minimize the caseinase production by *Aeromonas* spp. by affecting the enzymatic activity. This could support the possible relationship between salinity and a possible decrease of the protease activity by the ECP of Pdp11 in biofilm reduction. In any case, it would be interesting for future studies to investigate the potential involvement of enzymes, such as DNAse and/or quorum quenching activities in the reduction of the biofilm formation by these pathogens.

### Antiviral Effect of ECPs

This study has analyzed the potential effect of ECPs against the nervous necrosis virus (NNV), the causative agent of viral encephalopathy and retinopathy, a disease reported in more than 170 species of farmed and wild fish on all continents (Bandín and Souto 2020), and producing very serious economic losses in the aquaculture sector (Bandín and Souto 2020).

The present work demonstrates how the ECPs obtained from F and FS media and incubation at 15 °C during 24 and 48 h can interfere with the virus replication, while the ECPs from TSAs increased the viral titer. Maybe, this variability between F/FS media could be related to the anionic charge due to the presence of active and ionizable groups, as well as impredecible mineral content, that increase with the addition of salt. As it occurs with some supernatants and exopolysaccharides (EPS), these components could interfere with the absorption and penetration of viruses into host cells and inhibit retroviral reverse transcriptase activities (García et al. 2022). The findings of this study are consistent with those reported by Abdelhamid et al. (2019) on the antiviral potential of probiotic supernatants against the Newcastle disease virus (NDV) and infectious bursal disease virus (IBDV) in poultry. The authors attributed this activity to the production of antiviral metabolites which can reduce the pH (lactic acid) or interfere with virus adsorption to host cells (bacteriocins). This suggests that the antiviral activity could be produced by non-proteinaceous (e.g., hydrogen peroxide, lactic acid) and/or proteinaceous compounds (bacteriocins) (Al Kassaa et al. 2014), corroborating the importance of growing conditions in postbiotic potential.

Other authors describe that probiotics and its metabolites can interfere and protect against viral infections in different ways: (i) inhibition of several reverse transcriptases (Aghebati-Maleki et al. 2022), (ii) stimulating and enhancing the immunological response (Wu et al. 2020; Xie et al. 2022), (iii) enhancing gut immunity (Qiu et al. 2020; Chattaraj et al. 2022), (iv) blocking the viral binding to the host receptors (Kalinichenko et al. 2020), or (v) inducing the expression of *mx* gene in cells, which could inhibit the NNV RNA synthesis. In this way, Wu et al. (2020) isolated a strain of *Shewanella* genus from the intestine of the grouper that was able to produce metabolites showing activity against the NNV. These authors observed that the metabolites neither neutralized NNV nor blocked the viral receptor on GF-1 cells but induced the expression of the *mx* gene in cells, which could inhibit the NNV RNA synthesis. However, the specific mechanisms of ECPs of SpPdp11’s inhibitory effect on NNV are yet to be fully understood, and further experiments are necessary. Moreover, the next step could be directed to the use of bacterial extracts as adjuvants to vaccines. Currently, flagellin (Mizel and Bates 2010) and the use of outer membrane vesicles (OMVs) derived from certain bacteria (Mancini et al. 2020) have also been evaluated as an adjuvant for viral vaccines. In the future, postbiotics may also be explored as adjuvants, but their safety and efficacy must be deeply evaluated.

### Cytotoxicity of ECPs

The cytotoxic effect of any product should be tested before considering it as a potential candidate for any clinical or biological application (Di Nunzio et al. 2017). For this, the present study is crucial to accurate a better selection of postbiotics. In this regard, recently, it has been reported how postbiotics from *Lactobacillus casei* strains exerted an in vitro cytotoxic effect against colorectal cancer cells (Elham et al. 2022).

The results indicated a significant cytotoxic effect of F/FS internal controls regardless of temperature. The aquafeed used in this study includes compounds such as soybean concentrate and squid meal, commonly used in fish feed (Zhou et al 2018). Soybean concentrate has been reported to contain trypsin inhibitors and phytate, which may act as anti-nutritional factors by interfering with protein digestion or chelating nutritionally essential elements, including Ca, Zn, and Fe (Nile et al. 2020). Squid meal often contains heavy metals (Murthy et al. 2013) and mycotoxins that can induce oxidative stress leading to cytotoxic (Da Silva et al. 2018; Al-Ghafari et al. 2019; Branca et al. 2020). However, although these compounds are present in feed, they may not exert the same in in vivo experiments, where SpPdp11 has shown to confer beneficial effects on the host when administered through diet (Domínguez-Maqueda et al. 2021).

Despite the presence of these compounds, a reduction of the cytotoxic effect due to ECPs from F and FS media has been observed at 15 °C in FuB-1 and at 23 °C in PLHC-1, in comparison with their internal controls after incubation in F/FS media, which might suggest mitigation of the cytotoxicity by the ECPs from the probiotic bacteria. This finding could be attributed to the antioxidant properties (Esteban et al. 2014; Vidal et al. 2016) as well as the recognized capability of the *Shewanella* genus to remove contaminants such as heavy metals (Yuan et al. 2019; Misra and Ghosh Sachan 2022). Furthermore, the cytotoxic effect was found to be higher at higher concentrations, which agrees with previous results (Shahid et al. 2019) suggesting that the cytotoxic effects of a given compound could be dose-dependent. Although it may be challenging to increase the concentration of ECPs due to the standardized extraction method, future studies could evaluate additional doses to exclude any biphasic response.

Picot et al. (2004) reported that the cytotoxicity of *Pseudomonas fluorescens* is regulated by growth temperature, observing the highest cytotoxic effect of the bacteria at its optimum temperature of growth, probably by exerting an influence on the structure of proteins, whose cytotoxic effect could be modified (Nsonzi et al. 2015; Briaud et al. 2021). These findings are consistent with the present results as cytotoxicity was lower when ECPs were recovered at 15 °C than at 23 °C, despite 23 °C being the optimal growth temperature of SpPdp11. This highlights the importance of evaluating different growth conditions, such as temperature, in modulating the secretion of ECPs.

Of all cell lines, the PLHC-1 cell line showed the least cytotoxicity when exposed to ECPs, particularly those obtained from F medium and incubated at 15 °C. This cell line corresponds to fish hepatocytes, and the effect of ECPs on it may be related to the reported beneficial effect on the liver of farmed fish fed a diet supplemented with SpPdp11 cells (García de la Banda et al. 2010; Tapia-Paniagua et al. 2014c). In these mentioned works, the authors obtained liver microscopy images, and fish that received the Pdp11 diet showed significantly lower numbers of lipid droplets than those fed the control diet. In addition, hepatocyte lipid droplet size was smaller for Pdp11 specimens than those detected for fish control. These mechanisms are been investigated to know the molecules and routes implied. However, the study of different cell lines will allow us to select the best postbiotic candidates for further applications.

To conclude, the present work evaluates for the first time the postbiotic potential of ECPs secreted by the probiotic strain SpPdp11 when cultured under different growth conditions. These ECPs have been characterized on the basis of their in vitro hydrolytic, antagonistic, and antiviral capabilities, as well as their cytotoxic effect on different fish cell lines. The results obtained have shown the loss of some specific capabilities of ECPs present in living cells, such as antibacterial capacities. However, we can obtain interesting enzymatic activities, antiviral effects, inhibition of pathogen biofilm formation, and potential non-cytotoxic effects of some fish cell lines in in vitro assays. Moreover, depending on the different culture conditions of the probiotic strain, the obtention of the ECPs could be optimized to enhance each activity.

In conclusion, while the growth phase and culture media were important to achieve the desired results, NaCl supplementation led to a reduction in the potential postbiotic effect analyzed. The best overall results were obtained when grown in F medium at 15 °C (specifically F1524) followed by T medium at 23 °C (both T2324 and T2348). Due to the wide target points of postbiotics, their application could be very extensive to use in many industries, such as food, healthcare products, cosmetics, and nutraceuticals. In terms of the aquaculture industry, the optimized growth conditions can allow us to obtain promising postbiotics, which may be of interest for future in vivo experiments as supplemental additives for improving fish health.

### Supplementary Information

Below is the link to the electronic supplementary material.

Supplementary file1 (DOCX 14 KB)
