# Supplementary material for: Evaluation of the Differential Postbiotic Potential of Shewanella putrefaciens Pdp11 Cultured in Several Growing Conditions
Source: Mar Biotechnol (NY). 2023 Dec 28;26(1):1–18. doi: 10.1007/s10126-023-10271-y (PMC10869407; doi:10.1007/s10126-023-10271-y)
Supplement: Supplementary file 1 — Supplementary file1 (DOCX 14 KB) [file 10126_2023_10271_MOESM1_ESM.docx]

**Evaluation of the differential postbiotic potential of *Shewanella putrefaciens* Pdp11 cultured in several growing conditions.**

**Journal Research:** Marine biotechnology

Marta Domínguez-Maqueda^1^, Jorge García-Márquez^1^, Silvana T. Tapia-Paniagua^1,*^, Carmen González-Fernández^2^, Alberto Cuesta^2^, Cristóbal Espinosa-Ruíz^2^, Mª Ángeles Esteban^2^, FJ Alarcón^3^, Mª Carmen Balebona^1^ y Miguel Ángel Moriñigo^1^

^1^Departamento de Microbiología, Facultad de Ciencias, Instituto Andaluz de Biotecnología y Desarrollo Azul (IBYDA), Universidad de Málaga, Ceimar-Universidad de Málaga, Málaga, Spain; [martadm@uma.es](mailto:martadm@uma.es), [j.garcia@uma.es](mailto:j.garcia@uma.es), [stapia@uma.es](mailto:stapia@uma.es), [balebona@uma.es](mailto:balebona@uma.es), [morinigo@uma.es](mailto:morinigo@uma.es).

^2^Departamento de Biología Celular e Histología, Facultad de Ciencias, Universidad de Murcia, Murcia, Spain; [alcuesta@um.es](mailto:alcuesta@um.es), [carmen.gonzalez1@um.es](mailto:carmen.gonzalez1@um.es), [cer48658@um.es](mailto:cer48658@um.es), [aesteban@um.es](mailto:aesteban@um.es).

^3^Departamento de Biología y Geología, Universidad de Almería, Ceimar-Universidad de Almería, Almería, Spain; [falarcon@ual.es](mailto:falarcon@ual.es).

**Table S1.** Ingredient composition of the experimental diet used for the aquafeed culture media of ECP samples extraction. Aquafeed provided by Tecnovit Lifebioencapsulation

| **Composition** | **Content (%)** |
| --- | --- |
| Fish meal LT94 | 10,00 |
| Soycomil R | 15,00 |
| Wheat gluten | 17,00 |
| Pea protein concentrate | 5,00 |
| Soy meal (50%) | 20,00 |
| Wheat meal | 14,14 |
| Fish oil | 7,00 |
| Soy oil | 4,50 |
| Colza soil | 4,50 |
| Vitamins and minerals | 1,00 |
| Vitamin C | 0,05 |
| Vitamin E | 0,01 |
| Methionine | 0,50 |
| Monocalcium phosphate | 1,30 |
| Microalgaes |  |
| Summarized | 100,00 |
